# Impact of dementia care training on nurse care managers’ interactions with family caregivers

**DOI:** 10.1186/s12877-022-03717-w

**Published:** 2023-01-11

**Authors:** Taylor J. Mellinger, Brent P. Forester, Christine Vogeli, Karen Donelan, Joy Gulla, Michael Vetter, Maryann Vienneau, Christine S. Ritchie

**Affiliations:** 1 Mass General Brigham, Boston, USA; 2 Idaho College of Osteopathic Medicine, Meridian, USA; 3 Harvard Medical School, Boston, USA; 4 McLean Hospital, Belmont, USA; 5 Massachusetts General Hospital, Boston, USA; 6 Brandeis University, Waltham, USA

**Keywords:** Dementia, Care management, Family caregivers

## Abstract

**Background:**

Nurse care managers (NCM) operate through care management programs to provide care for persons living with dementia (PLWD) and interact regularly with their family caregivers; however, most do not receive formal instruction in dementia care or caregiver support. CRESCENT (CaReEcoSystem primary Care Embedded demeNtia Treatment) is a telephone-based dementia care intervention adapted from the Care EcoSystem model designed to equip NCMs with these tools. For this study, we aimed to measure intervention fidelity and understand how dementia care training impacted NCMs’ provision of dementia care management services during interactions with caregivers of PLWD.

**Methods:**

We recruited 30 active NCMs; 15 were randomly assigned to receive training. For each nurse, we randomly selected 1–3 patients with a diagnosis of dementia in each nurse’s care during January-June 2021 for a total of 54 medical charts. To assess training uptake and fidelity, we identified documentation by NCMs of CRESCENT protocol implementation in the medical records. To understand how the training impacted the amount and types of dementia care management services provided in interactions with family caregivers, we compared attention to key dementia topic areas between trained NCMs (intervention) and untrained NCMs (control).

**Results:**

Within the trained group only, community resources for PLWD, followed by safety, medication reconciliation, and advanced care planning topic areas were addressed most frequently (> 30%), while behavior management was addressed least frequently (12%). Trained NCMs were more likely to document addressing aspects of caregiver wellbeing (*p* = 0.03), community resources (*p* = 0.002), and identification of behavior (*p* = 0.03) and safety issues (*p* = 0.02) compared to those without training. There was no difference between groups in the amount of care coordination provided (*p* = 0.64).

**Conclusion:**

Results from this study demonstrate that focused dementia care training enriches care conversations in important topic areas for PLWD and family caregivers. Future research will clarify how best to sustain and optimize high quality dementia care in care management programs with special attention to the NCM-family caregiver relationship.

**Trial number:**

NCT04556097.

## Background

Accountable care organizations (ACOs) are groups of healthcare providers who work together to offer highly coordinated care to high-risk, high-need Medicare beneficiaries [1]. In the United States of America, ACOs have successfully lowered spending and acute care utilization through creation of integrated care management programs in which coordinated care is managed and provided by care managers, often nurses [2, 3]. Typical activities of such personnel include facilitating communication between providers, assisting with navigation of the healthcare system, and improving patient access to ensure that care is well coordinated [4]. While care management programs serve patients with an array of chronic conditions, they provide coordinated care to a meaningful subset of persons living with dementia (PLWD).

Family caregivers provide the bulk of home care to PLWD, an all-encompassing role which is often associated with high risks of depression, stress, anxiety, physical demands, and poor health outcomes [5]. The global cost of dementia is approximately $818 billion USD per year, approximately half of which is due to the cascade of supports needed for informal caregivers [6].

The roles of nurse care managers (NCM) and family caregivers intersect in the care of PLWD. Much of dementia care occurs in concert with the caregiver, whereas other disease states usually involve them more peripherally [7]. Additionally, as evidenced above, caregivers often need support for themselves. However, care managers are usually not provided with any formal training in dementia care or caregiver support [8], and nurses in our health system and in other care settings often report low efficacy in this area [9]. This gap leaves both NCMs and caregivers with little guidance to manage the complex roles for which many healthcare systems heavily rely on them.

To address this issue, we administered an adapted dementia care training to NCMs in our health system. This study sought to accomplish two aims. First, to assess the fidelity to specific training protocols among NCMs who received the adapted training. Implementation fidelity assesses whether or not an intervention is being adopted in alignment with the original intention, which is critical to understand before determining the intervention’s contribution to outcomes [10]. Our second aim was to understand the impact of dementia care training on NCMs’ documented interactions with caregivers. We hypothesized that NCMs who participated in the training program would demonstrate higher levels of fidelity to the intervention and would address issues related to dementia care, spanning beyond typical care coordination [4], in their documented interactions with caregivers. Importantly, we did not expect a difference in care coordination activities between groups as this is a core skill of all NCMs [8].

## Methods

### Care Ecosystem model

The Care Ecosystem (CareEco) model is a telephone and web-based collaborative dementia care intervention [11]. In its original form, trained, unlicensed dementia care guides called care navigators administered the intervention by providing support, education, and care coordination to PLWD and their caregivers. Care navigators were trained on seven specific dementia care protocols and received ongoing guidance from a nurse, social worker, dementia specialist, and pharmacist. With this model, the care team navigator was supported to address the complex needs of family caregiver patient dyads, and, as a result, caregivers were provided with personalized guidance and support [7]. Overall, the CareEco intervention improved quality of life for patients with dementia, bettered caregiver outcomes, and reduced emergency department visits [11]. Although CareEco clearly demonstrated value, not all healthcare systems routinely have care navigators integrated into their staffing models.

### Adaptation of Care Ecosystem for CRESCENT study

We adapted the CareEco model by engaging with NCMs already working with high-cost high-need patients in our health system and revising training and intervention materials to align with nursing scope of practice. The focus on upskilling care managers that were already working within our healthcare system can save costs by eliminating the need to hire new personnel to deliver dementia care. Adaptations were made to the materials with input from four senior nurse care managers over six hours of meetings. The six dementia care protocols utilized in training mirrored those of the Care Ecosystem intervention and included assessment of immediate needs and based on the assessment, addressing: (1) medication reconciliation and review, (2) behavior management, (3) safety assessment and counseling, (4) caregiver wellbeing assessment and counseling, (5) community resources/other referrals, and (6) decision-making evaluation/advance care planning [11]. We named the adapted intervention CRESCENT (CaReEcoSystem primary Care Embedded demeNtia Treatment).

CRESCENT training consisted of 60 min of e-learning modules and one day of live virtual learning delivered by a geriatrician and a geriatric psychiatrist who had received specific training in the original CareEco program. After training, the geriatrician and geriatric psychiatrist provided ongoing guidance through one hour per week of office hours and as needed email exchanges.

The intervention consisted of an initial needs assessment with the subsequent use of the six standardized protocols described above. Use of the protocols were individualized in that most urgent needs of PLWD-caregiver dyads were expected to be addressed first with review of subsequent topics occurring after the initial more urgent issues had been addressed. Phone calls to dyads were suggested to be monthly, but the frequency could be adjusted and updated according to caregivers’ preferences and current needs.

### Recruitment

Thirty practicing NCMs were recruited from the integrated care management program (iCMP) within a large integrated delivery system in Boston, MA containing two major tertiary care hospitals, five community hospitals, and more than 180 primary care practices [8]. All NCMs in our system are registered nurses. NCMs had general training around high-cost, high-need populations, but not specific to dementia care [8].

The NCMs recruited for this intervention were specifically identified because they had six or more months experience and worked within primary care practices with the highest proportion of patients who were from racial or ethnic minority groups or covered by Medicaid. Fifteen NCMs were randomized to receive training during the study, with the remainder eligible to receive training after the study’s conclusion. We randomly selected 1–3 patients in each nurse’s care with an ICD-10 diagnosis code consistent with dementia in Medicare claims in the six months following the intervention (January-June 2021) for a total of 66 patients. Charts were excluded if the patient died during the first five months of the study period, did not consent to medical record use for research, was a permanent resident of a skilled nursing or long-term care facility, was discharged from iCMP, or if there was no evidence of availability of a family caregiver to participate in the intervention. Chart review was restricted to documentation of care conversations with the family caregiver. We obtained patient demographic information from the electronic medical records.

The 180 primary care sites we recruited from were homogenous in that they were part of a specific health system in a specific region of the US. However, since we purposefully selected NCMs serving diverse sites, some practices may be more represented.

### Data collection and analysis

#### Fidelity analysis

To assess training uptake and implementation fidelity, we looked for documentation of CRESCENT protocol implementation in the free text of medical records documented by the NCMs who received training.

Our key variable of interest was the presence or absence of documentation of each of the six protocols. A protocol was counted as implemented if there was evidence of one or more subcomponent(s) of the six Care Ecosystem protocols described above and in Possin et al. [11] coupled with an action plan (for example, documentation of a behavioral symptom and a plan to change the patient’s schedule to ease agitation) in the care management notes at any time during the six month study period. We calculated the percentage of patients with protocol implementation according to the above definition. High frequency of protocol implementation helped indicate areas of fidelity to core components of the program, while low frequency of implementation identified gaps in training uptake and application.

#### Impact of intervention

To better understand the nature of specific dementia care topics that were addressed by NCMs and how NCMs worked with caregivers after CRESCENT training, we looked for evidence of each training protocol and each subcomponent [11] in both trained and untrained NCMs’ documentation of interactions with caregivers at any point during the six month study period. Authors CSR and TJM mapped data extracted from EHR to activities corresponding to each of six protocols and their subcomponents of the CRESCENT intervention for each patient record. To understand the impact of the training on conversations with caregivers, we compared the frequency of evident dementia care topics between the trained NCMs and the control group using chi squared and Fisher’s exact tests.

## Results

**Fig. 1:**
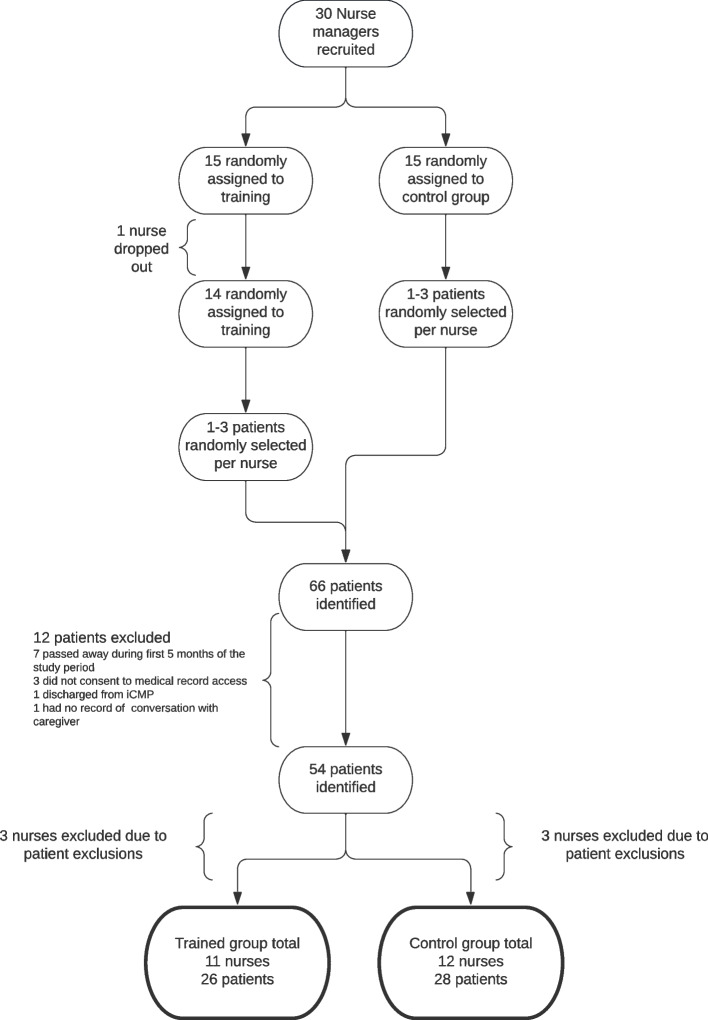
Recruitment flow

We originally selected 66 patients for the study; 12 were excluded by previously stated criteria, which led to the exclusion of six NCMs. Of the 54 patients studied, 26 were treated by 11 NCMs with training and 28 by 12 NCMs without training (Fig. 1).

As shown in Table 1, females were highly represented in this study sample (67%), with similar distributions in trained (69% female) and control groups (64% female). The average age was 85.5 years old, which was consistent throughout study arms. Most PLWD were white (83%), non-Hispanic (89%) and spoke English as their preferred language (85%).

**Table 1:** PLWD demographics

	Patients treated by trained NCMs *N* = 26	Patients treated by control NCMs *N* = 28	Total *N* = 54
Age (mean, sd)	85.5 (7.8)	85.5 (7.4)	85.5 (7.5)
Sex			
Female	18	18	36
Male	8	10	18
Race			
White	20	25	45
Asian	0	1	1
African American	1	0	1
Other	4	0	4
Declined	1	2	3
Ethnicity			
Hispanic	4	1	5
Non-Hispanic	22	26	48
Unknown	0	1	1
Language			
English	21	25	46
Spanish	5	1	6
Italian	0	2	2

**Fig. 2:**
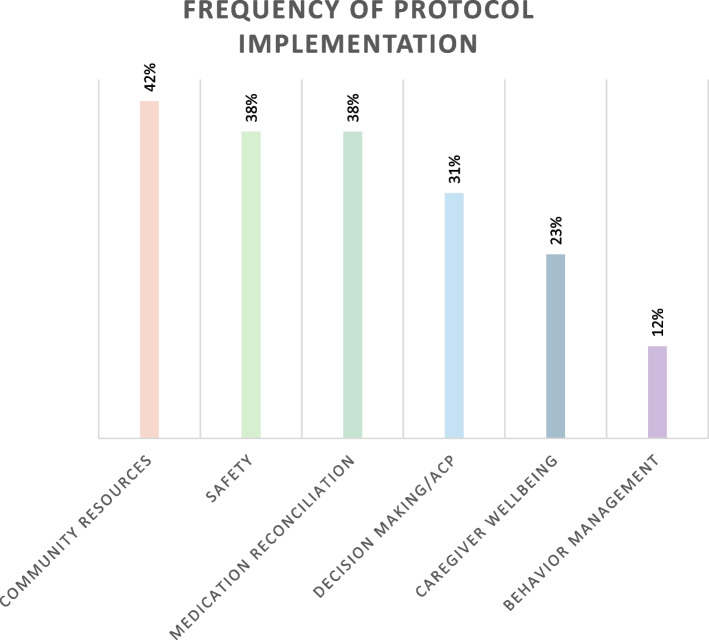
Legend: This figure shows frequency of protocol implementation by CRESCENT trained NCMs only. Data from 26 patient charts recorded by 11 trained NCMs

Results in this paragraph and Fig. 2 are from NCMs who received training only and therefore represent the uptake and fidelity of CRESCENT training. The community resources protocol was addressed most consistently, with 42% of medical records showing evidence of encouraging caregiver engagement with community-based programs such as adult day health programs, elder services, the Alzheimer’s Association, and caregiver support groups. This was closely followed by evidence of safety and medication reconciliation training. NCMs documented safety risks and provided personalized guidance to caregivers to promote PLWD’s safety and wellbeing in 38% of records reviewed. Medication reconciliation training was provided with the intent of the NCM acting as a core educator and risk manager for medication related issues and was also completed at a frequency of 38%. There was proof of identification of advance care planning needs, determination of capacity, and conversations regarding medical, legal, and financial planning, encompassing the advance care planning and decision-making protocol, in 31% of medical records. The caregiver well-being protocol showed a smaller uptake (23%) and included tasks such as utilizing caregiver assessment tools, providing support and education, and formulating plans to address caregiver wellbeing. Evidence of problematic behavior identification and management was seen least frequently (12%).

NCMs discussed medications and care coordination in most cases, regardless of training status. Likewise, nearly half of medical records among both groups addressed some aspect of caregiver wellbeing (Table 2).

**Table 2:** Key dementia care areas addressed by trained versus control NCMs

	Records by trained NCMs (*n* = 26)	Control (*n* = 28)	Chi-square/Fisher *p*-value
Any subcomponent of caregiver wellbeing protocol	14 (53.8%)	12 (42.9%)	0.42
Navigating multiple caregivers	9 (34.6%)	3 (10.7%)	**0.03**^**a**^
Caregiver financial wellbeing	3 (11.5%)	0	0.10
Caregiver physical health	4 (15.4%)	1 (3.6%)	0.13
Safety risk identification	7 (27%)	1 (3.6%)	**0.02**^**a**^
Safety management	3 (11.5%)	0	0.10
Identify backup caregiver	3 (11.5%)	0	0.10
Preferences for future medical care	4 (15.4%)	1 (3.6%)	0.13
Any subcomponent of behavior protocol	16 (61.5%)	8 (28.6%)	**0.01**^**a**^
Behavior identification	15 (57.7%)	8 (28.6%)	**0.03**^**a**^
Behavior management	3 (11.5%)	0	0.10
Community resources	13 (50%)	3 (10.7%)	**0.002**^**a**^
Any subcomponent of medication protocol	15 (57.7%)	16 (57.1)	0.97
Care coordination	18 (69.2%)	21 (75%)	0.64

Legend: Numbers of patient medical records that showed evidence within the 6-month study timeframe of addressing key topics important for dementia care derived from subcomponents of CRESCENT training based on CareEco protocols [11]. Numbers of records with documentation of dementia care topics was compared between patients treated by NCMs who received training and those in the control group

Bolded values with ^a^ represent a significant difference between groups in whether the topic was addressed

NCMs who completed CRESCENT training were more likely to address key topics associated with high quality dementia care than NCMs that had not completed training. The largest difference between groups was seen in provision of and referrals to community resources, with 50% of medical records of PLWD in the trained group showing evidence of conversations in this area versus only 10.7% in the control group (*p* = 0.002). Trained NCMs also addressed behavioral signs and symptoms with greater proportions of PLWD and their caregivers (*p* = 0.01), although the difference was only in identification of behavioral issues (*p* = 0.03), rather than management of them (*p* = 0.1). Conversations and documentation about safety followed a similar pattern with NCMs in the trained group having a 7-fold increase in identifying safety issues but not a significant difference in formulating safety plans. Medical records of patients treated by NCMs in the trained group were significantly more likely to include evidence of conversations regarding elements of caregiver well-being, specifically navigation of multiple caregivers (stresses of sharing care responsibilities and engaging other caregivers for respite and care coverage) (*p* = 0.03). NCMs who completed training were more likely to address preferences for future medical care in their conversations with dyads, however this difference did not reach significance (*p* = 0.13). As expected, there was no difference between groups in frequency of care coordination (*p* = 0.64).

## Discussion

Community resources training showed the greatest uptake in our fidelity analysis as well as the greatest difference in being addressed in conversation and documentation when comparing trained and control NCMs. It could be that some aspects of the education and coordination components involved in the community resources protocol were similar to tasks that NCMs were already preforming. Therefore, NCMs successfully incorporated the protocol into their daily tasks when they were trained on various resources available for PLWD and caregivers. Utilization of community resources has been shown to increase caregivers’ confidence and support in caring for PLWD [12].

The behavior management training showed the smallest uptake at 12% of records showing evidence of core protocol components and action plans. However, in comparison to NCMs who had not completed training, trained NCMs showed a significant increase in their identification of behavior issues. Behavioral symptoms in dementia are commonplace, with a recent study finding the prevalence of depression, apathy, and agitation/aggression to each be over 30% [13]. For this reason, the difference in behavior identification between groups is likely not due to a difference in occurrence of behavioral symptoms but to increased attention to the symptoms by the NCM. This improvement not only provides an opportunity for NCMs to better manage these complications with supervision and further training, but also to refer to specialists for the most challenging behaviors. Additionally, misunderstanding the meaning of behaviors has been identified as a key theme contributing to caregivers’ challenges with behavioral symptoms [14]. Naming and understanding the cause of a behavior may aide the caregiver in coping and approaching challenges at home. The difference between groups was not seen when it came to management of these behaviors. The complexity of understanding the causes of behavioral symptoms in dementia and the application of specific pharmacological and non-pharmacological therapies is challenging, time consuming, and new to NCMs. Furthermore, fidelity analysis review only occurred in the first six months after training; discussing and addressing behavioral issues might take more time to navigate. Regardless, behavior management is made possible by behavior identification and therefore we believe this to be a step forward in this aspect of dementia care.

Conversations with caregivers about safety followed a similar story, with significantly more records written by trained NCMs showing evidence of identifying safety risks but not of safety planning. Therefore, the management aspect of both behavior and safety could be an area to expand training or provide more consultation with dementia care specialists. Of note, we also observed a trend of trained NCMs being more likely to identify a back-up caregiver. Alternate caregivers are crucially important for safety, but a suggestion like this could also ease caregiver burden and strengthen caregivers’ perception of support.

The decision making and advance care planning protocol showed an uptake of 31% within trained NCMs. This protocol required understanding of when to initiate the advanced care planning conversation, expertise to identify current plans and potential needs, and knowledge to provide appropriate resources and referrals. The breadth of medical, legal, and financial planning questions allows for comprehensive care planning; however, the detailed nature could also be a barrier to completing the protocol for either the NCM or the caregiver-patient dyad. Advance care planning also has an emotional element that some families or NCMs may not be prepared to address [15]. Because these conversations may take more time and careful effort, it may not be realistic to assess the implementation of this protocol in a six month period.

The caregiver wellbeing training was implemented at a lesser rate of 23% but did show promising indications of recognizing the importance of the care partner in comprehensive dementia care. As mentioned previously, most care management works directly with patients. However, due to changing decision-making capacity and nature of Alzheimer’s disease and related dementias, the caregiver is a critical effector of care coordination in dementia care. Although studies have shown poor outcomes on mental and physical health for caregivers [16] as well as greater self-efficacy among caregivers who have more support [17], a successful method to include them in a dementia care model has not been widely implemented. The CRESCENT caregiver wellbeing training focused on screening for caregiver strain, asking empathic questions, engaging in collaborative goal setting, and providing resources. The lower protocol implementation could be related to NCMs prioritizing more medically pressing and time sensitive issues, so something that seems less urgent could be delayed. It is also possible that caregivers were not as interested in engaging in conversations about themselves, and perhaps wanted to focus on issues directly related to the PLWD. Interestingly, trained NCMs were more likely to document when there were multiple caregivers involved. Access to caregiver contact information is often unclear from the medical record or other documentation available. Sharing caregiving responsibility, which is quite common in dementia care, can complicate this further. We also observed a trend in increased discussions regarding caregiver financial wellbeing and caregiver physical health. These are promising steps toward recognizing care partners as important members and receivers of dementia care.

There were no differences between the trained and untrained groups in discussing topics related to medications. Medication reconciliation and management is a core function of many high-risk care management programs [8]. Because of this, we would not necessarily expect to see differences in implementation of the medication management protocol between the trained and control groups. However, tailoring training to cover specific medications and management issues of PLWD, such as attending to medication anticholinergic burden, may enhance care. We also did not observe a difference between groups in conversations and documentation of care coordination. This is also expected as NCMs see care coordination as a core part of their job.

Given the diverse and personalized needs of PLWD and family caregivers, more care may not necessarily mean better care. However, CareEco has shown improved outcomes including increased quality of life for PLWD, better caregiver outcomes, and decreased emergency department visits by incorporating dementia care training protocols [11, 18]. Therefore, we believe that adherence to a great number of training protocols in CRESCENT may yield similar outcomes representing high quality dementia care.

As far as we know, this is the first intervention with a focus on training already existing NCMs in a primary care system to deliver dementia specific care. In a narrative review of dementia care models within primary care, nurses, social workers, or unlicensed dementia care managers were utilized primarily for care coordination and were not provided with training in dementia care [19]. Other studies have explored the benefits of providing dementia care training to personnel such as primary care physicians, inpatient staff, and home care workers [20–22].

Furthermore, our study is unique in that we assessed documentation of interactions between NCMs and caregivers to better understand the effect of our training intervention. As our healthcare system relies on these individuals heavily in the context of dementia care, future studies are needed to expand our understanding and optimization of the NCM-family caregiver partnership.

This study was limited in its recruitment and care of a racially and ethnically diverse patient population. Despite choosing primary care practices with greater racial and ethnic diversity prior to randomization, most NCMs and PLWD were still white, non-Hispanic, and English speaking. Notably, 14.8% spoke a language other than English. Further, the study period was limited to six months, and it is possible that greater or different effects would be observed over a longer observation period. Additionally, due to the short time period, we cannot comment on the sustainability of our training intervention. Finally, as we were relying on the documentation of clinical encounters, it is possible that some conversation topics were missed if they were not documented in the medical records.

## Conclusion

We assessed intervention fidelity of a dementia care training program and the training’s effects on the amount and types of dementia focused care provided by NCMs during interactions with family caregivers. We hypothesized that NCMs who completed training would be more likely to address key dementia care topics and would show no difference in amount of care coordination provided.

Most of the dementia care protocols showed a moderate to good uptake in a short period of time (six months). As evidenced by differences between the intervention and control group and previous results from the CareEco model, the training influenced more comprehensive assessment and management of important dementia care topics. As NCMs provide a large amount of dementia care and coordination in healthcare systems and family caregivers provide the bulk of care at home, the interaction between the two is paramount. With training, the role of the NCM can expand to not only provide comprehensive dementia care to the PLWD, but also to offer individualized support to the family caregiver. Wide adoption of dementia care training for NCMs could have far reaching positive effects on NCM self-confidence in their roles, caregiver wellbeing, and functionality of sustainable dementia care models.

## Data Availability

The data for this study was abstracted from patients’ medical record and is not publicly available. To request data from this study, please contact the corresponding author (Taylor Mellinger).

## Abbreviations

ACO: accountable care organization
NCM: nurse care manager
PLWD: persons living with dementia
CRESCENT: CaReEcoSystem primary Care Embedded demeNtia Treatment
CareEco: Care Ecosystem
iCMP: Integrated Care Management Program
