# Correction: Impact of dementia care training on nurse care managers’ interactions with family caregivers

**DOI:** 10.1186/s12877-023-03827-z

**Published:** 2023-03-30

**Authors:** Taylor J. Mellinger, Brent P. Forester, Christine Vogeli, Karen Donelan, Joy Gulla, Michael Vetter, Maryann Vienneau, Christine S. Ritchie

**Affiliations:** 1 Mass General Brigham, Boston, USA; 2 Idaho College of Osteopathic Medicine, Meridian, USA; 3 Harvard Medical School, Boston, USA; 4 McLean Hospital, Belmont, USA; 5 Massachusetts General Hospital, Boston, USA; 6 Brandeis University, Waltham, USA

**Correction: BMC Geriatrics 23, 16 (2022)**

**https://doi.org/10.1186/s12877-022-03717-w**

After publication of this article [1], the authors reported that in the Funding information section the following was missing: “This work was funded by the National Institute on Aging (NIA) of the National Institutes of Health under Award Number U54AG063546, which funds NIA Imbedded Pragmatic Alzheimer’s and AD-Related Dementias Clinical Trials Collaboratory (NIA IMPACT Collaboratory). The content is solely the responsibility of the authors and does not necessarily represent the official views of the National Institutes of Health.’

The original article [1] has been corrected.
